# Enhanced phoxim biodegradation by immobilizing *Novosphingobium* sp. RL4 on attapulgite-sodium alginate

**DOI:** 10.3389/fmicb.2025.1541328

**Published:** 2025-04-10

**Authors:** Tong Peng, Yining Huang, Tao Yang, Yinquan Wang, Ling Jin

**Affiliations:** ^1^Basic Medical Research Centre, School of Medicine, Nantong University, Nantong, China; ^2^College of Pharmacy, Gansu University of Chinese Medicine, Lanzhou, China; ^3^Key Laboratory of Microbial Resources Exploitation and Application, Institute of Biology, Gansu Academy of Sciences, Lanzhou, China

**Keywords:** phoxim, *Novosphingobium* sp., sodium alginate, attapulgite, biochar

## Abstract

**Background:**

Residual phoxim pollution presents a potential threat to natural ecosystems and human health. The immobilization of degrading strains on natural adsorbent materials is a common strategy to enhance the degradation of target compounds in the environment by the strains.

**Methods:**

A phoxim-degrading bacterial strain was isolated from the rhizosphere soil of rhubarb (*Rheum palmatum* L.), which had been exposed to long-term phoxim contamination. To enhance its stability and practical applicability, sodium alginate (SA) was utilized as a carrier material, while biochar (BC) and attapulgite (ATP) served as adsorption materials. These components were used to immobilize the strain, forming three distinct bacterial bead formulations: SA-RL4, SA + BC-RL4, and SA + ATP-RL4.

**Results:**

The isolated phoxim-degrading strain was identified as *Novosphingobium* sp. RL4. Furthermore, the degradation products of phoxim by strain RL4 were analyzed and characterized. Based on the specific surface area, mass-transfer performance results, adsorption isotherms, and degradation efficiency, the addition of ATP or BC to SA has an equally positive impact on the degradation of phoxim by immobilized microspheres. ATP can replace BC as an adsorbent carrier material for embedding bacteria to a certain extent. At 20 mg/L, SA + ATP-RL4 degraded 89.37% of phoxim in 72 h. Importantly, SA + ATP-RL4 can be reused, and the degradation efficiency remained above 80% after 5 cycles. Furthermore, it exhibits high tolerance and better degradation ability compared to free cells of RL4 when used in treating agricultural wastewater containing phoxim.

**Conclusion:**

SA + ATP-RL4 shows potential for *in situ* remediation of phoxim-contaminated environments.

## 1 Introduction

Phoxim is a widely used broad-spectrum organophosphorus insecticide, and its main action involves the inhibition of acetylcholinesterase activity. This leads to the accumulation of acetylcholine in the postsynaptic membrane and ultimately the death of pests (Lionetto et al., 2013). In China, 1,000 tons of phoxim are used in agriculture and fishing every year. It can be used to control many harmful insects and underground pests by dipping, spraying, or pouring, which has a positive impact on crop and fishery production (Huang et al., 2013). Phoxim is sensitive to light and easily decomposes under conditions of high temperature and sufficient light. However, it has a relatively long half-life in soil with a maximum of 49.5 days (Liu et al., 2015), which could potentially lead to residue accumulation with widespread and prolonged use. Rain erosion can also transport residual phoxim from soil into water bodies (Chao and Chen, 2014; He et al., 2016). The use of this they neurotoxic compound (Eevers et al., 2017) can have deleterious effects on non-target organisms, including mammals and aquatic species, ultimately affecting the environment and human health. Therefore, addressing the environmental and food safety risks associated with the application of phoxim is of paramount importance.

Bacterial bioremediation is widely considered to be both cost-effective and effective for the management of pesticide residues (Cycoń et al., 2017; Gangola et al., 2022). Studies have shown that the microbial resources involved in phoxim degradation are predominantly bacterial species, including *Stenotrophomonas* sp. SMSP-1 (Shen et al., 2010), *Stenotrophomonas* sp. G1 (Deng et al., 2015), and *Cupriavidus nantongensis* X1T (Shi et al., 2019). However, exogenous bacterial strains often struggle to effectively compete with indigenous microorganisms, making it difficult for them to colonize and survive in contaminated environments, thereby limiting their degradation efficiency. Therefore, the development of locally adapted functional bacterial strains is essential (Piccardi et al., 2022). Furthermore, research indicates that the degradation efficiency of free bacterial strains is often suboptimal, possibly due to their sensitivity to changes in environmental physicochemical parameters (e.g., pH, temperature, nutrient levels, and pollution concentrations), which results in reduced survival rates of free bacterial strains (Stelting et al., 2014).

In recent years, microbial immobilization techniques have been increasingly applied in bioremediation due to their ability to enhance microbial resistance and improve degradation efficiency (Dzionek et al., 2016). The primary microbial immobilization methods include adsorption, entrapment, and covalent bonding. The adsorption method utilizes adsorbents to attach pollutant-degrading bacterial strains. For instance, Xiong et al. (2017) reported that immobilizing *Mycobacterium gilvum* on rice straw biochar (BC) significantly enhanced its phenanthrene removal efficiency by 32.35% compared to using *M. gilvum* alone. However, cells immobilized on adsorbent carriers are prone to detachment during application (Jin et al., 2017). In contrast, entrapment and covalent bonding methods can effectively prevent the leakage and detachment of degrading bacterial strains. In particular, the entrapment method offers several advantages, including increasing microbial density, enhancing bacterial activity, ensuring long-term storage stability of immobilized beads, and improving the adaptability of bacteria to soil and aquatic environments in agricultural systems (Nadaroglu et al., 2019). Recent research has focused on incorporating adsorption materials into embedding materials to enhance the degradation of functional strains by effectively capturing pollutants. These two materials exhibit a synergistic effect in bioremediation applications (Fang et al., 2021; Wang et al., 2014).

Sodium alginate (SA) is one of the most widely used bacterial entrapment carriers, attributed to its low cost, non-toxicity, high biomass loading capacity, and excellent biocompatibility (Ouyang et al., 2021). Fang et al. (2021) successfully integrated 3% SA with various adsorptive materials, including diatomite (KLG), chitosan (CTS), and polyvinyl alcohol (PVA), to immobilize functional bacterial strains for chlorpyrifos degradation, yielding promising results. However, compared to natural adsorbents, these synthetic materials are associated with higher costs and lower microbial compatibility. BC is widely used as a natural adsorbent due to its high porosity, large surface area, and strong adsorption capacity, enabling the enrichment of multiple pollutants and microorganisms (Rajapaksha et al., 2015; Lawal et al., 2019; Patel et al., 2021). However, its production requires high pyrolysis temperatures, resulting in significant environmental and economic costs. To reduce these impacts, it is crucial to explore sustainable, low-carbon, and cost-effective natural adsorbents as alternatives or supplements to BC. Attapulgite (ATP), a clay mineral in the bentonite family, consists of hydrated magnesium silicate with a layered chain-like structure (Dai et al., 2022; Zghair et al., 2022). Its distinctive rod-like morphology and porous structure confer exceptional adsorption properties. China possesses abundant ATP reserves, particularly in Linze County, Gansu Province, with an estimated deposit of 400 million tons and a potential reserve of up to 1 billion tons. Given its superior adsorption characteristics, ATP presents significant potential for applications in agricultural environmental remediation (Hu et al., 2022).

In this study, phoxim-degrading bacterial strains were isolated from farmland soils with long-term phoxim contamination. To enhance degradation efficiency, the strains were immobilized using carrier materials composed of sodium alginate (SA) alone or in combination with attapulgite (SA + ATP) or biochar (SA + BC) as adsorptive components. The objectives of this study are: (1) to isolate and identify phoxim-degrading bacterial strains and analyze the degradation products of phoxim; (2) to evaluate the immobilization efficiency of different carrier materials for phoxim-degrading bacteria and identify the most suitable carrier; and (3) to elucidate the synergistic effects between carrier materials and adsorbents in phoxim degradation.

## 2 Materials and methods

### 2.1 Chemical reagents

Acetonitrile [high-performance liquid chromatography (HPLC) grade] was purchased from MREDA (Beijing, China). Phoxim chemical standard with purity of 99.5% was purchased from Dr. Ehrenstorfer GmbH (Augsburg, Germany). ATP was obtained as a suspended powder from Gansu Rongwan Technology Co., Ltd. (Gansu, China). It was screened through a 300-mesh sieve and stored. The measured pH was 8.9.

BC was produced by pyrolyzing corn straw at 500°C for 2 h under oxygen-free conditions. The BC was then crushed, sieved (300 mesh), and used in experiments. The BC had an organic carbon content of 42.21%, total nitrogen content of 8.34%, total phosphorus content of 2.31%, total potassium content of 16.12%, ash content of 7.23%, and pH of 9.46. Scanning electron microscopy (SEM) images of the ATP and BC are shown in Supplementary Figure S1. The mineral salt medium (MSM) contained 1.32 g/L K_2_HPO_4_·3H_2_O, 0.3 g/L KH_2_PO_4_, 0.1 g/L MgSO_4_·7H2O, and 1.0 g/L NaCl, and its pH was 7.0. Luria-Bertani (LB) medium contained 1.0% NaCl, 0.5% yeast extract, and 1.0% peptone, with a pH of 7.0.

### 2.2 Isolation, cultivation, and identification of strain RL4

The strain *Novosphingobium* RL4 was isolated from the rhizosphere soil of rhubarb (*Rheum palmatum* L.) in Hexi Town, Huating City, Gansu Province, which was contaminated with phoxim. The strain RL4 was cultured in MSM containing 5 mg/L phoxim at 28°C with shaking at 150 rpm for 5 days. The process was repeated using higher concentrations of phoxim (10, 20, 50, and 100 mg/L) until a pure strain was obtained. The purified strain was routinely subcultured on MSM plates supplemented with 20 mg/L phoxim.

Strain RL4 was cultured in LB at 28°C with shaking at 180 rpm until reaching an OD_600_ of 0.8 (logarithmic growth phase). The cells were then harvested by centrifugation at 5,000 rpm for 10 min, washed twice with sterile aqueous solution, and prepared for further experiments.

The bacterial morphology was observed using scanning electron microscopy (SEM; Tescan CLARA, Czech Republic). The 16S rRNA gene of strain RL4 was sequenced by Beijing Qingke Biotechnology Co., Ltd., and the obtained sequences were submitted to the NCBI GenBank database. Homology analysis and online sequence alignment were conducted using BLAST, and closely related sequences were selected for phylogenetic analysis. A phylogenetic tree was constructed using the neighbor-joining method in MEGA 11 software (Kumar et al., 2018). Strain RL4 has been deposited in the China General Microbiological Culture Collection Center (CGMCC) under accession number CGMCC No. 29226.

### 2.3 Analysis of phoxim degradation products by RL4

To analyze the metabolic products of phoxim degradation by the strain RL4, a 3% (v/v) bacterial suspension was inoculated into 20 mL of MSM medium containing 20 mg/L phoxim. The cultures were incubated at 28°C with shaking at 150 rpm, and samples were collected at 6-h intervals. The reaction was terminated by adding 20 mL of acetonitrile, followed by centrifugation at 12,000 × g for 10 min. The supernatants were filtered through a 0.22 μm membrane filter before chromatographic and mass spectrometric analysis. Chromatographic separation was performed using a Thermo Scientific^™^ Accucore^™^ aQ C18 polar end-capped LC column (10 cm length, 2.1 mm inner diameter, 2.6 μm particle size) with octadecylsilane-bonded silica gel as the stationary phase. The mobile phase consisted of acetonitrile (A) and 0.1% formic acid solution containing 5 mmol/L ammonium formate (B), and gradient elution was conducted as follows: time (min) = 0-10-14-14.1-20, with mobile phase A = 20-80-80-20-20. The flow rate was set at 0.2 mL/min, and the column temperature was maintained at 40°C. Mass spectrometric detection was performed using a triple quadrupole tandem mass spectrometer (ESI-QqQ-MS) equipped with an electrospray ionization (ESI) source in positive ion scanning mode, with the following parameters: spray voltage of 3,200 V, capillary temperature of 300°C, sheath gas flow rate of 40 Arb, auxiliary gas flow rate of 8 Arb, maximum spray current of 100 μA, and heated probe temperature of 300°C.

### 2.4 Preparation of immobilized beads

Free wet cells of strain RL4 were obtained using the activation culture method described in section 2.2. For the preparation of SA-RL4, 2% (w/v) wet cells of strain RL4 and 3% (w/v) sodium alginate (SA) were suspended in a sterile aqueous solution. The mixture was then added dropwise into a 4% CaCl₂ solution to form SA-RL4 immobilized bacterial beads. For SA + ATP-RL4, 2% (w/v) wet bacterial cells of strain RL4, 3% (w/v) SA, and 1% (w/v) attapulgite (ATP) were combined in a sterile aqueous solution. The solution was dropwise introduced into 4% CaCl₂, resulting in SA + ATP-RL4 immobilized bacterial beads. Similarly, for SA + BC-RL4, 2% (w/v) wet cells of strain RL4, 3% (w/v) SA, and 1% (w/v) biochar (BC) were mixed in a sterile aqueous solution, then dropwise added into 4% CaCl₂ to produce SA + BC-RL4 immobilized bacterial beads.

### 2.5 Characterization of immobilized beads

#### 2.5.1 Viability of immobilized bacteria during storage

The immobilized strains were stored at 4°C, and samples were collected every 7 days. To assess bacterial viability, 1 g of immobilized pellets was suspended in 10 mL of 0.6 mol/L trisodium citrate, followed by shaking extraction at 28°C and 180 rpm for 2 h. The extracted solution was then serially diluted, plated on LB medium, and incubated for 2 days before colony counting.

#### 2.5.2 Mass transfer and mechanical strength

The mass-transfer efficiency was evaluated by adding immobilized strains for vibration adsorption, which resulted in a decrease in the absorbance of methylene blue (Jin et al., 2017). One gram of immobilized microspheres was added to an 800 mg/L methylene blue solution and shaken at 28°C and 150 rpm for 3 h. We measured the absorbance of the supernatant at 665 nm before and after the oscillation adsorption of fixed microspheres and calculated the difference. We determined the proportion of complete gel beads through a vibration test to evaluate the mechanical strength (Chen and Lin, 2007). We placed 100 fixed bead samples into 250 mL of sterile water and shook them at 150 rpm and 28°C for 48 h. We calculated the amount of undamaged fixed beads as a percentage of all beads. All treatments were performed in triplicate.

#### 2.5.3 Structural characterization

After freeze-drying, the microstructure and surface elements of the materials were detected using cold field emission SEM (Tescan CLARA, Czech). The surface area of immobilized beads was measured using the Brunauer–Emmett–Teller (BET) method (BELSORP-mini II, Microtrac BEL, Japan). The functional groups of RL4, SA, ATP, BC, and immobilized bacterial beads (SA-RL4, SA + ATP-RL4, and SA + BC-RL4) were determined by Fourier-transform infrared spectroscopy (FTIR) (INVENIO S, Brooke, Germany). The samples were freeze-dried and made into a powder. Subsequently, 1-mg powder samples were mixed with 100 mg of KBr, ground into particles, and analyzed in the spectral range of 4,000 to 400 cm^−1^.

### 2.6 Adsorption kinetics of phoxim on carrier materials

SA, SA + ATP, and SA + BC immobilized beads were introduced into a 20 mL phoxim solution (20 mg/L) and incubated at 25°C with continuous shaking at 150 rpm in the dark. Samples were collected at 2, 6, 12, 24, and 48 h to analyze the adsorption kinetics of phoxim on the immobilized carriers. The residual phoxim concentration was quantified using HPLC. All treatments were conducted in triplicate. The adsorption capacity (*Q*_CP_) of phoxim on the three carrier materials was calculated using Equation 1.


(1) $$Q_{CP}=V\left(C_{0}-C_{T}\right)/m$$

In this equation, *C*_0_ and *C*_T_ are the initial and final concentrations of phoxim, respectively, *V* is the volume, and *m* is the mass of the immobilized beads.

### 2.7 Degradation kinetics of phoxim on immobilized beads

A total of 1 g of immobilized beads (SA, SA + ATP, or SA + BC) or 0.02 g of free bacteria was introduced into 20 mL of phoxim solution (20 mg/L) and incubated in the dark on a rotating shaker at 150 rpm and 28°C. Samples were collected at 0, 2, 6, 12, 24, 48, and 72 h, and the reaction was terminated by adding 20 mL of acetonitrile. All experiments were conducted in triplicate. The degradation kinetics of phoxim were analyzed by quantifying its concentration at different time points.

### 2.8 Reusability and stability of SA + ATP-RL4

A total of 1 g of SA + ATP-RL4 immobilized bacterial microspheres was added to 20 mL of MSM solution containing 20 mg/L of phoxim and incubated at 150 rpm and 28°C in the dark for 72 h. After incubation, the immobilized bacterial particles were removed, and the reaction was terminated by adding 20 mL of acetonitrile. The particles were then reintroduced into a fresh 20 mL MSM solution with 20 mg/L of phoxim, and the process was repeated until the degradation efficiency of phoxim by the immobilized bacterial beads declined to below 80%.

### 2.9 Phoxim degradation in agricultural wastewater

The agricultural wastewater used in this study was collected from irrigation drainage in a concentrated cultivation area at the Huating Rhubarb Plantation Field, Gansu Province, China (106.557432°E, 35.337197°N). We adjusted the pH to 7.2 to minimize the impact of pH on phoxim hydrolysis. Next, we measured the degradation rate of phoxim by adding 20 mL of agricultural wastewater with a phoxim concentration of approximately 10 mg/L and either 10^8^ CFU of free bacterial cells of strain RL4 (equivalent to approximately 1 g of immobilized beads) or 1 g of fixed RL4 bacterial beads in a conical flask.

A treatment with non-inoculated RL4 was used as a control. SA + ATP-RL4 was incubated in a rotating shaker at 150 rpm and 28°C in the dark for 72 h. Samples were collected periodically, and an equal amount of acetonitrile was added to stop the reaction. All treatments were performed in triplicate.

### 2.10 Phoxim analysis

An Agilent HPLC system with an Agilent Zorbax C18 HPLC column (2.1 mm × 100 mm, 1.7 μm) was used to analyze the content of phoxim. Phoxim can be separated at 30°C using an isogradient procedure according to the instructions for the determination of phoxim content from Ehrenstorfer GmbH (Augsburg, Germany). The mobile phase consisted of 20% water (A) and 80% acetonitrile (B). The detection wavelength was 205 nm, and the sample size was 10 μL.

### 2.11 Statistical analyses

The data was analyzed using Analysis of Variance (ANOVA) in SPSS 17.0 for Windows (IBM SPSS Inc., Chicago, United States), and the results are expressed as the mean ± standard deviation. A *p*-value less than 0.05 suggested significant differences.

## 3 Results

### 3.1 Identification of strain RL4

**Figure 1:**
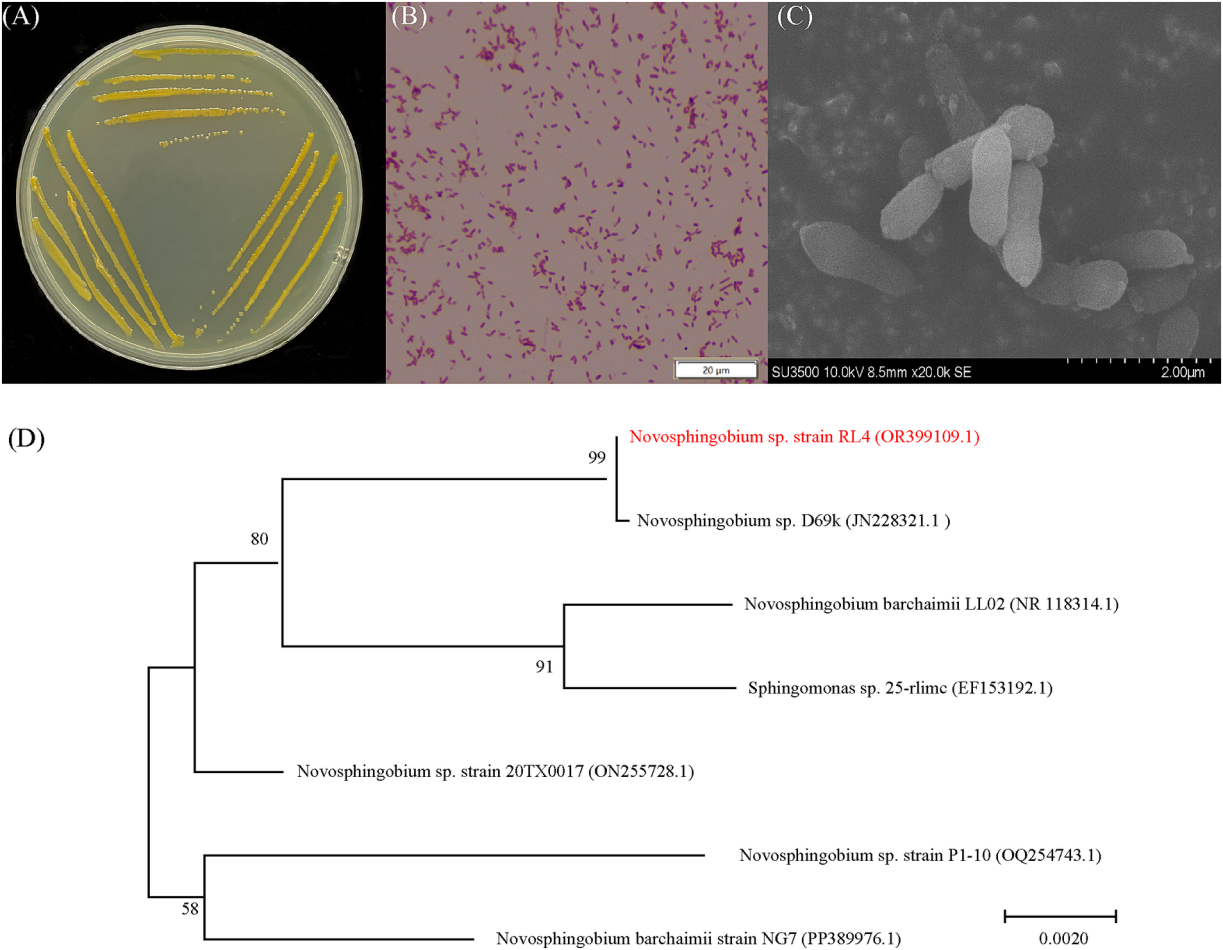
Morphology and identification of strain RL4. **(A)** A typical colony of strain RL4 grown on a LB-agar culture plane. **(B)** Gram staining image of strain RL4. **(C)** Scanning electron microscope image of strain RL4. **(D)** Phylogenetic tree of the 16S rRNA genes of strain RL4 and related bacteria constructed using the neighbor-joining method in MEGA 7.0 software.

The isolated RL4 strain exhibited growth on LB, nutrient broth, nutrient agar, and MSM containing phoxim as the sole carbon source. Following incubation at 28°C on LB plates for 48 h, a single colony displayed a diameter of approximately 2 mm and appeared moist, yellow, round, and raised with smooth edges (Figure 1A). Strain RL4 was identified as a Gram-negative bacterium with a short rod-shaped morphology lacking endospores or flagella (Figure 1B). Its cells measured around 1.5 μm in length and 0.8 μm in width (Figure 1C). The strain also had the highest pairwise similarities with *Novosphingobium* sp. D669k (JN228321.1) based on 16S rRNA sequences (Figure 1D). Therefore, the isolated strain was identified a *Novosphingobium* sp.

### 3.2 Analysis of phoxim degradation products by RL4

**Figure 2:**
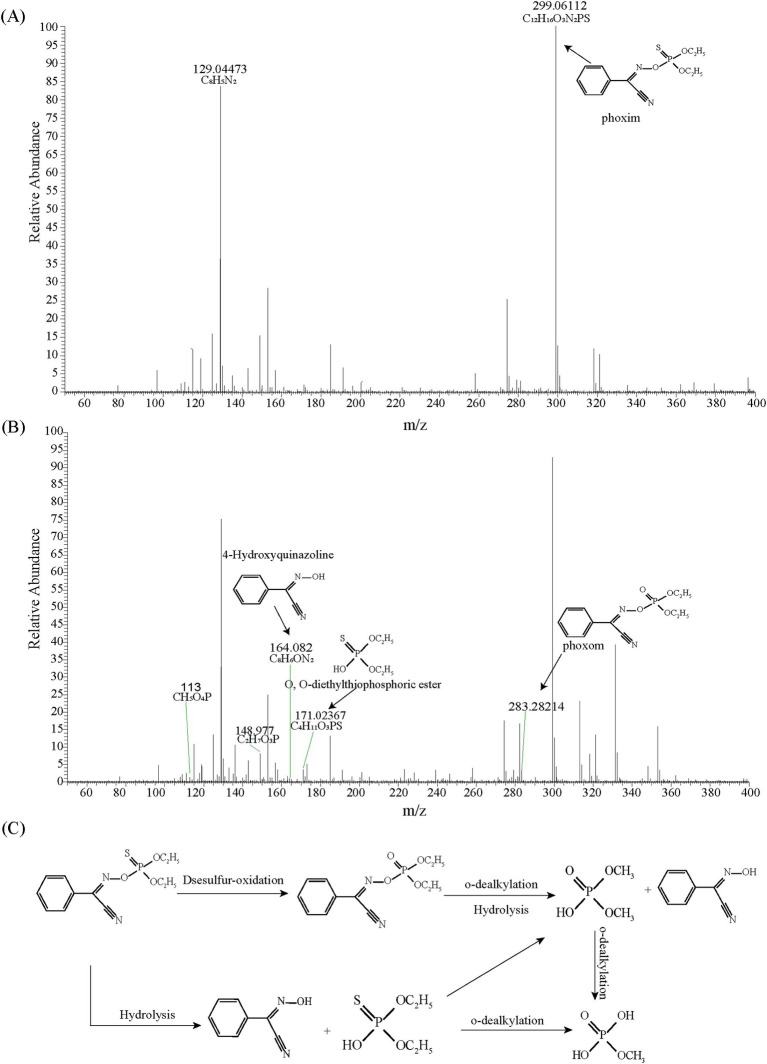
Mass spectra of the metabolic products of phoxim degradation by strain RL4 at 0 h **(A)** and 6 h **(B)**, and the proposed metabolic pathway of phoxim by strain RL4 **(C)**.

According to the HPLC-MS analysis, four phoxim metabolites were identified as methyl dihydrogen phosphate, phoxom, O,O-diethylthiophosphoric ester, and 4-Hydroxyquinazoline (Figures 2A,B). Based on these findings, a proposed metabolic pathway for phoxim degradation by strain RL4 is illustrated in Figure 2C. Strain RL4 degrades phoxim via two distinct pathways: (1) hydrolysis of phoxim to 4-hydroxyquinazoline and O,O-diethylthiophosphoric ester, followed by progressive dealkylation to form phosphate compounds; (2) oxidative desulfurization of phoxim to phoxom, which subsequently undergoes o-dealkylation, hydrolysis, and further dealkylation to produce phosphates for microbial utilization. However, the observed phoxim degradation pathway in this study appears incomplete, which may be attributed to the selected degradation time points.

### 3.3 Characterization of immobilized beads

#### 3.3.1 Storage viability, mass transfer, and mechanical strength

**Figure 3:**
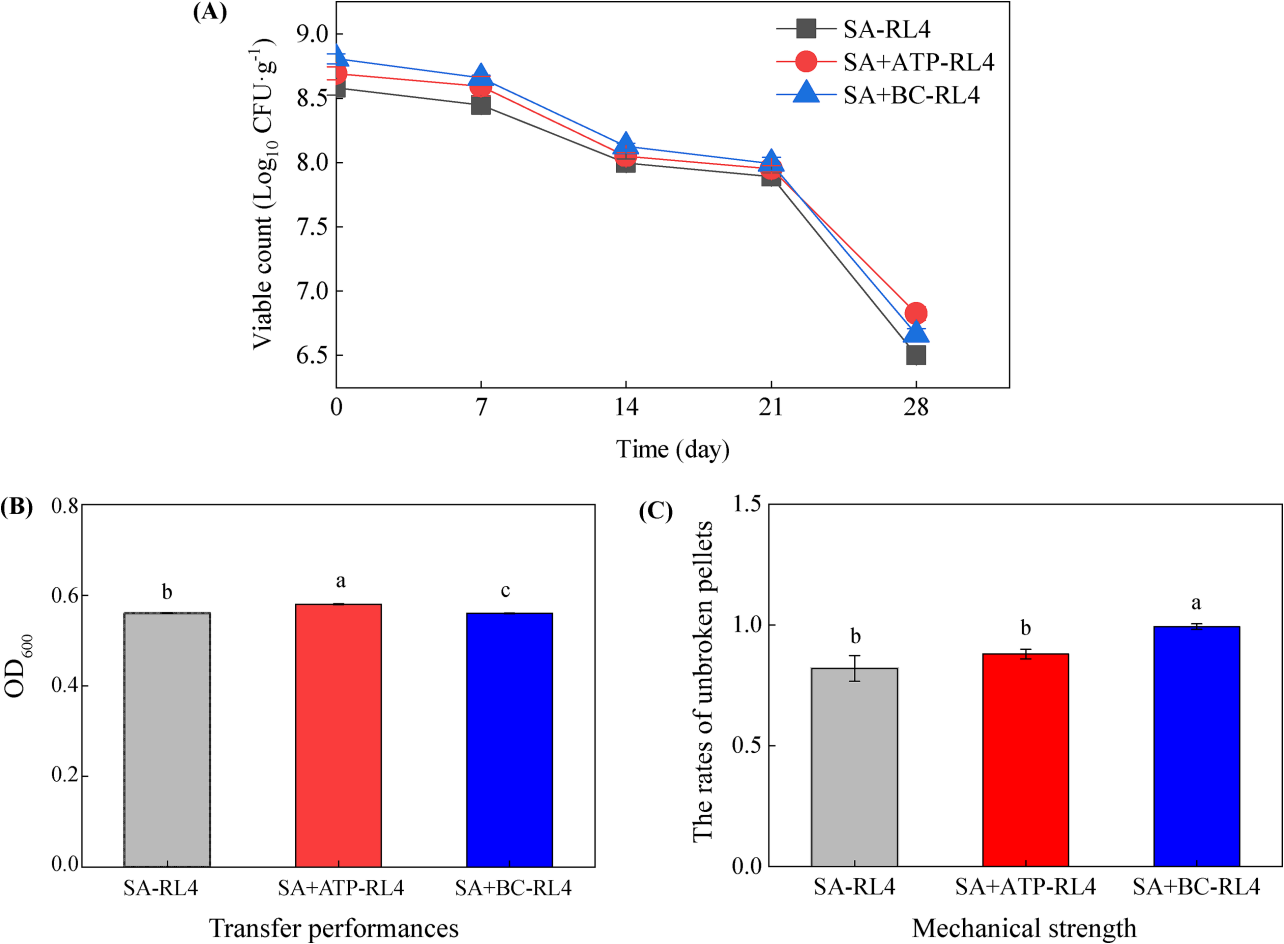
The number of viable bacteria **(A)**, mass transfer performance **(B)**, and mechanical strength **(C)** of three types of immobilized beads. Data are the means of three replicates with standard deviations (±SD). Different lower-case letters indicate significant differences among treatments (one-way ANOVA, *p* < 0.05).

The numbers of viable *Novosphingobium* RL4 cells embedded in SA/SA + ATP/SA + BC were 8.58, 8.69, and 8.81 log_10_ CFU/g, respectively. The number of viable bacteria embedded with the three immobilization methods decreased with the storage time (Figure 3A). After 28 days of storage, the cell numbers of RL4 embedded in SA, SA + ATP, and SA + BC decreased by 24.3, 22.5, and 23.3%, respectively. The immobilized bacteria demonstrated good viability (greater than 75% survival) after 28 days of storage at 4°C, which most likely resulted from the low temperature inhibiting microbial metabolism. The diameters of three types of immobilized beads ranged from 3.5 to 5.0 mm (Supplementary Figure S2). The mass-transfer performance was measured as 0.56 to 0.58, and SA + ATP-RL4 exhibited the highest performance (Figure 3B). The mechanical strength also ranged from 0.56 to 0.58, and SA + BC-RL4 showed the highest value (Figure 3C).

#### 3.3.2 SEM

**Figure 4:**
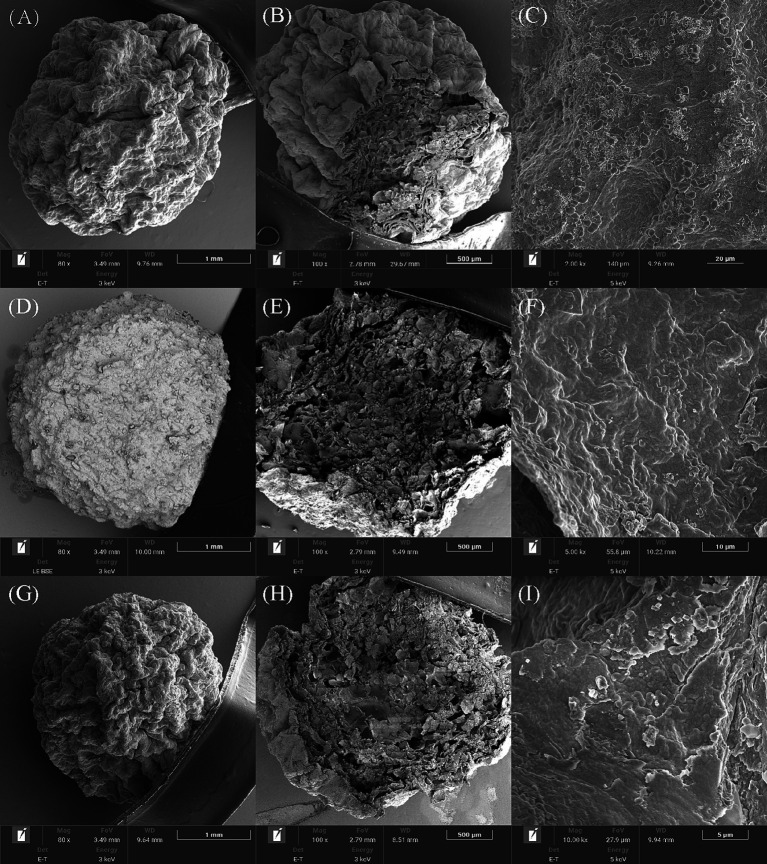
Scanning electron microscopy (SEM) analysis of three types immobilized beads. SA-RL4 **(A–C)**, SK-RL4 **(D–F)** and SP-RL4 **(G–I)**.

As shown in Figure 4, the addition of ATP rods and BC improved the surface formation and support of bacterial beads after freeze-drying. BC had the best effect and presented a dense spherical shape. The interior of the three kinds of immobilized bacteria beads showed honeycomb structures and contained multiple cavities, which provided sufficient space for the adhesion and proliferation of microorganisms. Compared to SA alone, adding adsorbent materials BC and ATP increased the internal space. The SEM results were similar to those reported by previous studies, in which bacteria colonize the surface of BC materials by forming cell aggregates similar to biofilms (Wang et al., 2019). Based on the embedding efficiency and 28-day survival rate, all three embedding materials effectively enhance strain density while preserving biological activity.

#### 3.3.3 BET analysis

The BET data further illustrate the structural differences of the immobilized beads (Table 1). Compared with SA-RL4, the addition of BC and ATP resulted in 2.1-fold and 2.2-fold increases in the specific surface area, respectively. Compared with SA, the mean pore diameter and total pore volume of SA + BC were reduced by 55.99 and 64.19%, respectively. Notably, SA + ATP exhibits high specific surface area, total pore volume, and average pore size.

**Table 1:** Brunauer–Emmett–Teller (BET) characteristics of four types immobilized beads.

Treatment	BET surface area (m^2^/g)	Total pore volume (cm^3^/g)	Mean pore diameter (nm)
SA	0.2016	0.002477	49.1586
SA-BC	0.6289	0.00109	17.606
SA-ATP	0.6374	0.00309	47.847

SA, sodium alginate; BC, biochar; ATP, attapulgite.

#### 3.3.4 FTIR

**Figure 5:**
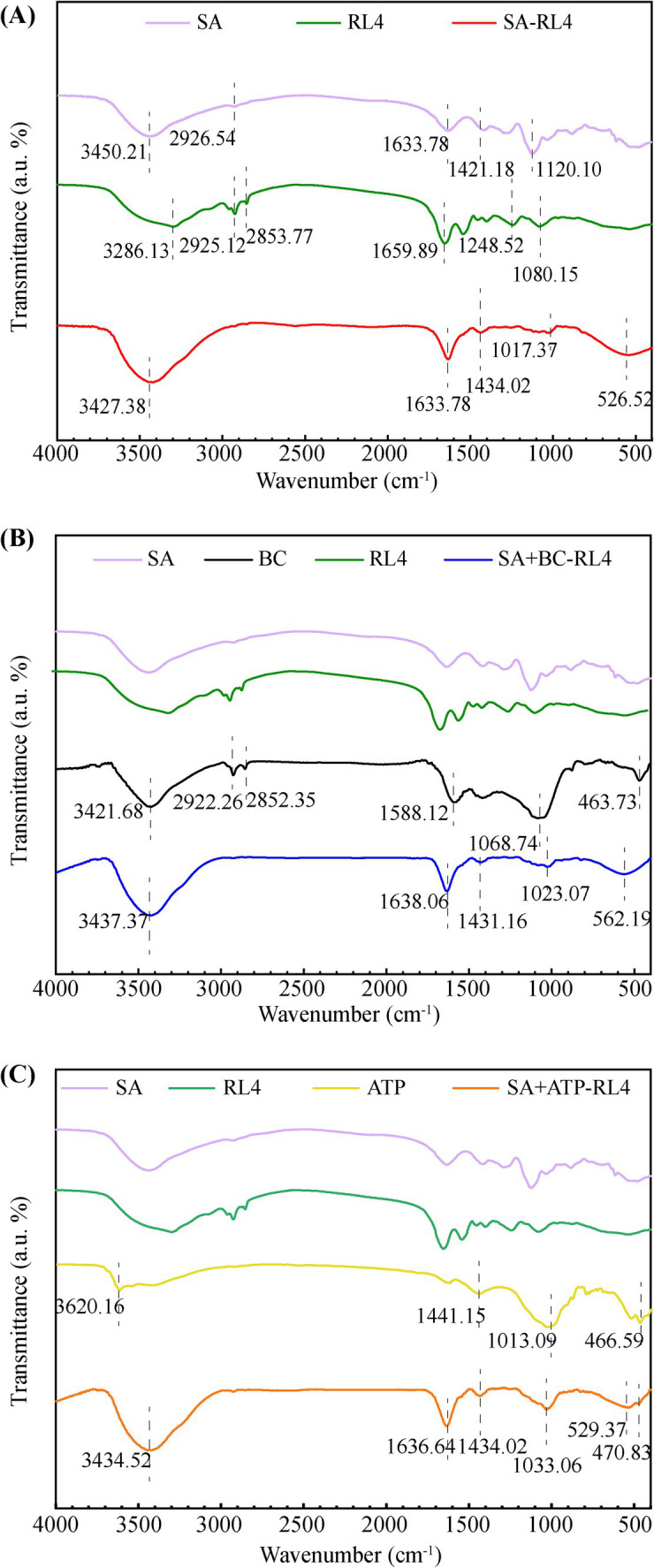
Fourier-transform infrared spectroscopy (FTIR) spectra of three types immobilized beads. SA-RL4 **(A)**, SA + BC-RL4 **(B)**, and SA + ATP-RL4 **(C)**.

In the infrared spectrum of BC (Figure 5B), characteristic peaks of hydroxyl group (–OH) and aliphatic group (C–H) occurred at 3,421 cm^−1^, 2,922 cm^−1^, and 2,852 cm^−1^, and a characteristic peak of aromatic C=C stretching occurred at 1,588 cm^−1^. The peak at 1,068 cm^−1^ indicated carbonates of C–O–C and C–O groups (CO_3_^2−^), while the peak near 460 cm^−1^ is associated with Si–O–Si tensile vibration. Studies have shown that hydroxyl is derived from cellulose, hemicellulose, and lignin in raw BC materials, and Si–O–Si functional groups are derived from silicon in BC. Characteristic bands of ATP occurred at 3620, 1441, 1,058, and 463 cm^−1^ and correspond to Al–OH, –OH, Si–O–Si, and O–Si–O, respectively (Figure 5C). These peaks resulted from the Al and Si in ATP.

Among the three types of immobilized bacterial beads, the stretching vibration peak of the fatty C-H bond formed by RL4 at 2,925–2,928 cm^−1^ was no longer present (Huang et al., 2015), and all exhibited characteristic vibration peaks at 3420.25–3433.09, 1633.78–1635.21, 1434.16–1434.02, and 1025.93–1028.78 cm^−1^ (Figures 5A–C). Notably, the immobilized strains demonstrated higher peak strength at 3420.25–3433.09 cm^−1^, indicating the presence of tensile vibrations from multiple hydroxyl groups in the material binding. COO^−^ symmetric and asymmetric tensile peaks formed at 1,618 cm^−1^ and 1,421 cm^−1^, which indicate enhanced carboxyl group complexation between materials.

Asymmetric stress peaks around 1017.37–1033.06 cm^−1^ indicate Si–O–Si bonding, and the characteristic peak of SA + ATP-RL4 shifted to higher wavenumber, demonstrating its strong affinity for Si–O–Si bonds. Both SA-RL4 and SA + ATP-RL4 showed broad Si–OH–Si peaks near 529 cm^−1^, while SA + ATP-RL4 produced characteristic peaks of O–Si–O at 460 cm^−1^. Different from SA-RL4, the characteristic peak generated by SA + BC-RL4 at 562 cm^−1^ may be attributed to the vibration of Fe–O bonds (Açin Ok and Kutluay, 2023).

### 3.4 Adsorption and degradation kinetics

**Figure 6:**
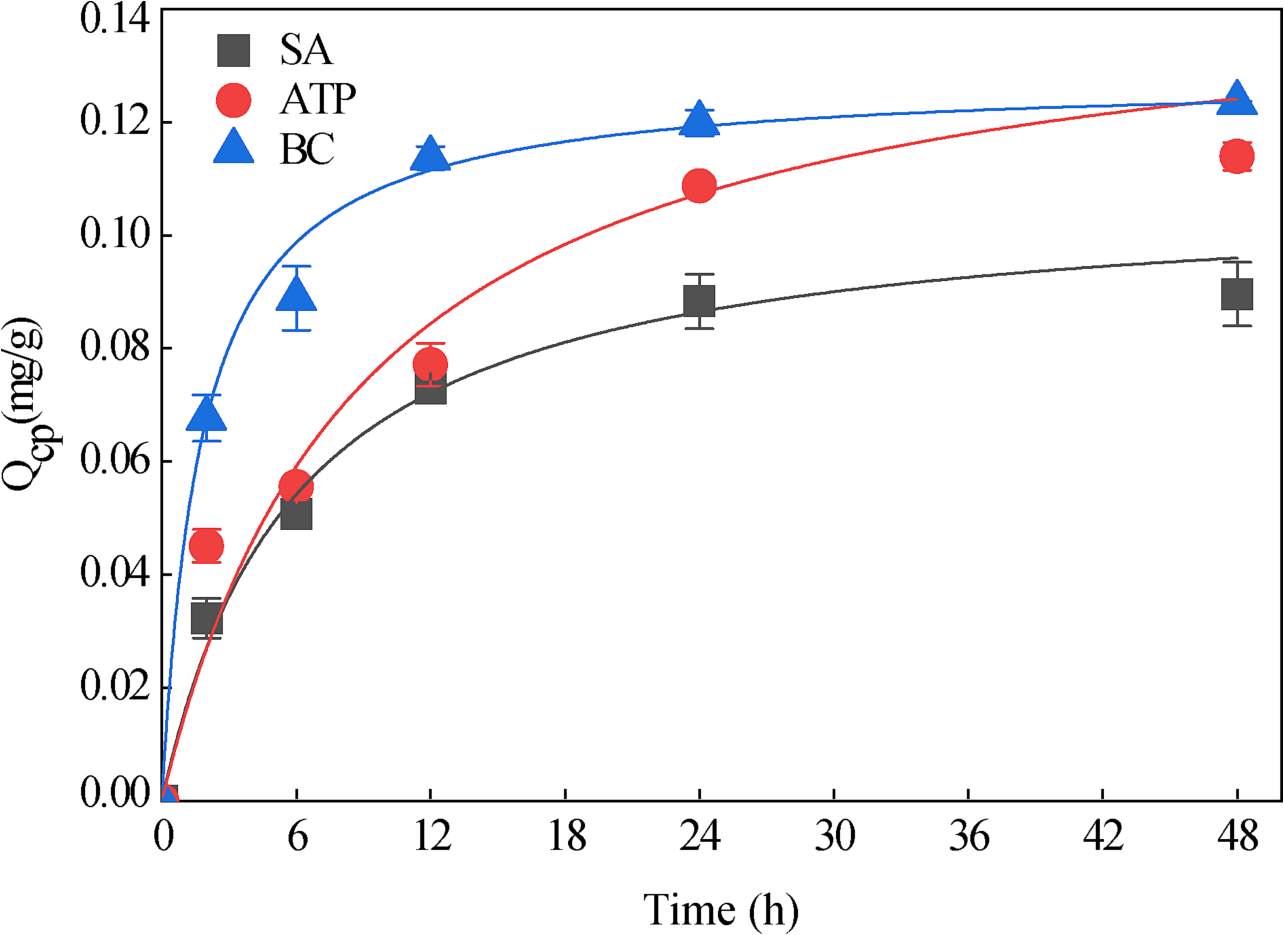
Adsorption equilibrium of 20 mg/L phoxim by three types immobilized beads without strain RL4. Data are the means of three replicates with standard deviations (±SD).

**Figure 7:**
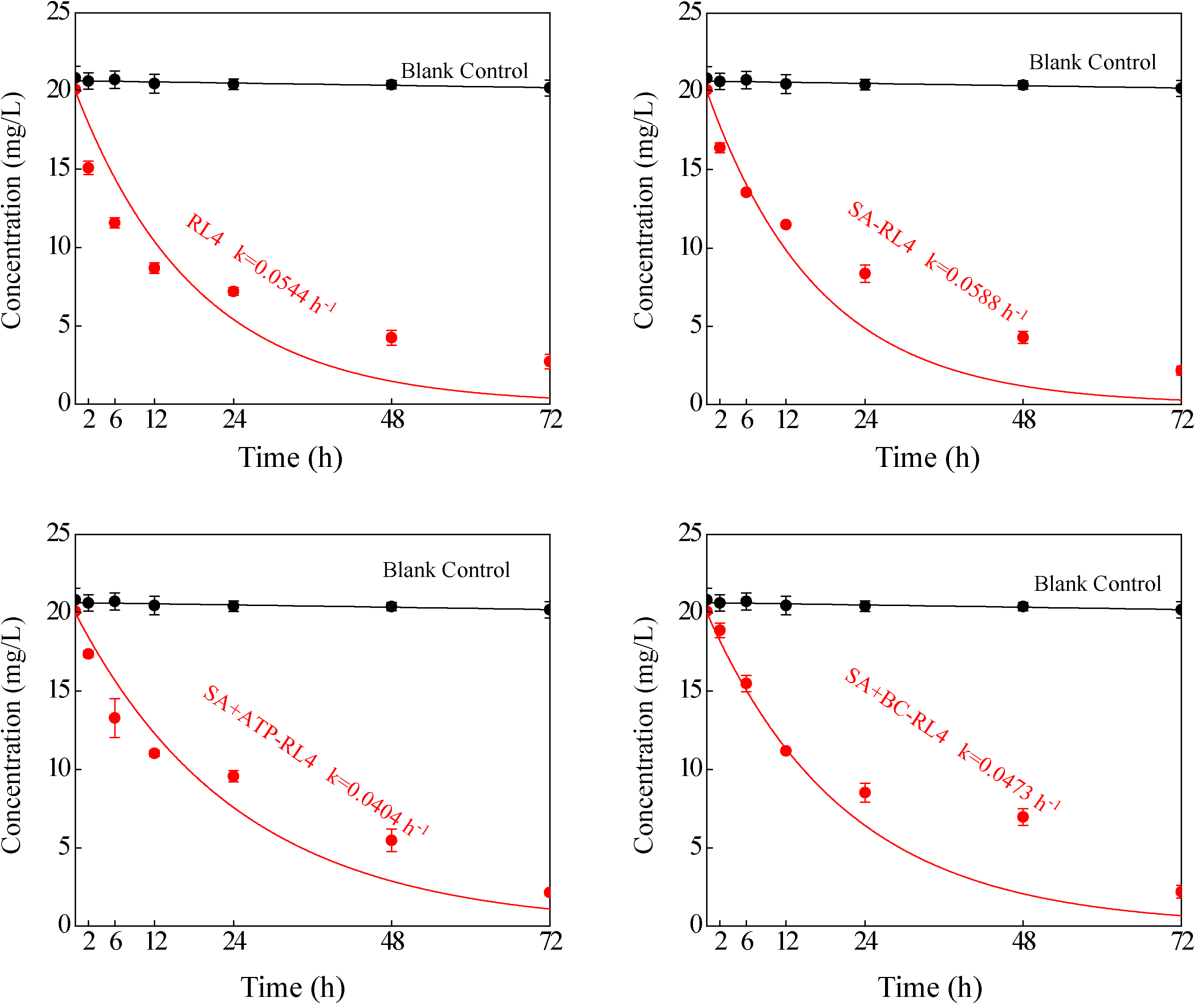
Degradation kinetics of 20 mg/L phoxim by RL4, SA-RL4, SA + ATP-RL4, and SA + BC-RL4. Data are the means of three replicates with standard deviations (±SD).

The maximum adsorption capacity of SA was 0.089 mg/g, that of SA + BC was 0.113 mg/g, and that of SA + ATP was 0.124 mg/g. The three immobilized strains were all fitted well with the Langmuir equation (SA: *R*^2^ = 0.97; SA + BC: *R*^2^ = 0.98; SA + ATP: *R*^2^ = 0.95; Figure 6). The degradation kinetics of 20 mg/L phoxim for the free strain RL4 and three types of immobilized beads (Figure 7). SA-RL4, SA + ATP-RL4, and SA + BC-RL4 demonstrated phoxim degradation rates of 86.76, 87.70, and 88.39% within a 72-h period, and their corresponding half-lives were 13.15, 17.01, and 14.51 h, respectively.

### 3.5 Reusability of immobilized beads and phoxim degradation in polluted agricultural water

**Figure 8:**
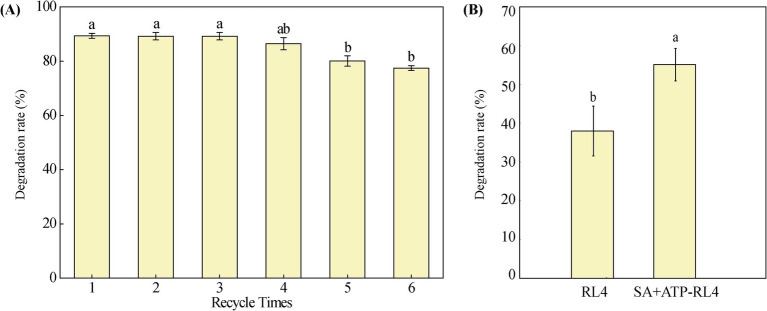
Reusability of SA + AT-RL4 for phoxim degradation **(A)**, and degradation of phoxim in agricultural polluted water by RL4 and SA + AT-RL4 **(B)**. Data are the means of three replicates with standard deviations (±SD). Different letters indicate significant differences among treatments (one-way ANOVA, *p* < 0.05).

After immobilization on SA + ATP, RL4 consistently demonstrated 80% degradation of phoxim even after undergoing 5 cycles of reuse (Figure 8A). This suggests the practical feasibility of reusing immobilized strain SA + ATP-RL4 for *in situ* remediation of phoxim (Shen et al., 2020). The degradation rate of phoxim at 72 h was analyzed in polluted agricultural water. The rate of free cells was only 37.96%, but the rate of SA + ATP-RL4 reached 55.11%, indicating better tolerance of wastewater (Figure 8B).

## 4 Discussion

Functional microbial bioremediation strategies are increasingly used for soil or water contaminated with OPPs (Chen et al., 2016; Shi et al., 2019). We have presented a biological improvement strategy for cleaning agriculturally contaminated water containing phoxim, which involved screening and identifying phoxim-degrading bacteria followed by microbial immobilization. The functional strain was identified as a species of *Novosphingobium*. Members of this genus are frequently found in polluted environments, including those with organic phosphorus contamination, and show promising potential for bioremediation. The investigation of organophosphorus pesticide degradation pathways and metabolites revealed that phoxim-degrading bacteria primarily utilize a series of hydrolysis, desulfurization-oxidation, and dealkylation reactions to convert phoxim into bioavailable phosphorus, supporting their growth. Similar to the degradation metabolism of phoxim observed by Meng et al. (2019) using *Bacillus amyloliquefaciens* YP6, this study identified additional intermediate metabolites involved in the dealkylation process, providing further insights into the microbial degradation mechanism.

Studies have shown that BC concentration in the range of 1–3% is positively correlated with the mass-transfer efficiency of immobilized particles of immobilized strains (Kou et al., 2023). However, high concentrations of BC can reduce the bioactivity of immobilized particles and reduce their degradation efficiency. We chose 1% BC for further study (0.3 g of beads containing 0.006 g of BC), which was based on the degradation of phoxim mainly being affected by microorganisms rather than BC, as well as the single-factor experiment on BC addition conducted by Wang et al. (2018). According to reported research results, when 1% ATP is added to SA, immobilized microspheres can maintain their adsorption capacity, improve the mechanical strength of SA, and reduce preparation costs. As the ATP content increases, the adsorption capacity decreases. The addition of 1% ATP concentration to SA is also within the optimal concentration range of 0.25–2% according to a previous study (Wang et al., 2014). For comparability with BC, the addition amount of concave convex rods was also set at 1%.

Compared with SA alone, the composite carrier SA + BC/ATP can improve the stability and capacity of immobilized beads. All three embedding materials have the capacity to increase the density of strain RL4 and preserve its biological activity. However, BC or ATP added to SA can play the role of a supporting skeleton in the immobilized bacterial beads, improve the mechanical strength and mass-transfer performance, and make the dense gel structure become rough and porous. Thus, the strain RL4 can more easily adhere to and multiply on these carriers, which is consistent with previous studies (Lu et al., 2021). Strain RL4 exhibited a higher survival rate when SA + ATP was used as a composite carrier compared to SA + BC. This difference may be attributed to the formation of a honeycomb structure by BC, where larger pore sizes result in smaller pore volumes. The addition of BC led to a more compact surface of the immobilized pellets, which did not enhance mass-transfer performance or support the proliferation of microorganisms within the pellets.

The SA + BC/ATP composite carriers can improve the adsorption capacity of the immobilized microspheres. The adsorption kinetics data fit well with the Langmuir model, indicating that the immobilized microspheres adsorbed phoxim in a single layer with uniform distribution of adsorption sites (Zhou et al., 2014; Kong et al., 2019). It has been widely recognized that the specific surface area, porosity, and functional groups of immobilized microspheres are important for improving the adsorption capacity of target pollutants (Meng et al., 2021). The fibrous morphology, large specific surface area and pore size, abundant oxygen-containing functional groups (–OH and Al–OH), and the presence of silico-oxygen bonds (such as O–Si–O and Si–O–Si) in ATP can create more active sites for the adhesion of organic pollutants (Meng et al., 2021). SA-RL4, SA + ATP-RL4, and SA + BC-RL4 have similar functional groups, including –OH bonds, C–H, and COO^−^. These functional groups participate in surface ion exchange, cationic −*π* bonds, and coordination complexation, which are potential adsorption mechanisms for exogenous substances. They can be combined with polysaccharides and protein residues on the surface of degraded strains to enhance the stability of immobilized strains (Zhang et al., 2020). Compared with SA, SA + ATP-RL4 has stronger Si–O–Si binding ability and has special O–Si–O bonds. Unlike SA, SA + BC-RL4 has Fe–O bond vibration. These functional groups can enhance the adsorption of organic phosphorus (Ahmed et al., 2018; Zhu et al., 2018).

Compared to SA alone, adding ATP as a composite carrier can also improve the degradation ability of immobilized beads. It has been shown that the adsorption and degradation capacities of the immobilized beads are synergistic. The addition of ATP improved the adsorption and degradation rate of phoxim by immobilized bacterial beads, and the effect was comparable to that of BC. SA + ATP-RL4 showed better degradation than the free strain RL4 in agricultural wastewater. In general, the composition of agricultural wastewater is complex, which can reduce the activity of degrading strains and affect their function. Consistent with the immobilized strains *Rhodococcus* sp. KLW-1 (Kou et al., 2023) and *Phanerochaete chrysosporium* (Huang et al., 2015), the immobilized strain SA + ATP-RL4 can still maintain 80% phoxim degradation activity after 5 repeated uses, indicating that it is feasible for *in situ* remediation of phoxim. The main reason is that ATP is a nutrient carrier with multiple pores that can provide sufficient space to maintain bacterial growth.

In summary, microbial immobilization is a promising bioremediation strategy in agriculture, but its efficiency depends on carrier materials, microbial adaptability, immobilization methods, and environmental conditions. In this study, the SA + BC/ATP composite carrier effectively enhanced the adsorption of phoxim while maintaining microbial activity, reinforcing the synergistic relationship between adsorption and degradation in the immobilization of the phoxim-degrading strain RL4. Key challenges in sustainable agricultural management include variability in field conditions (e.g., soil type and pollutant levels), toxicity of degradation byproducts, and the biosafety of functional strains, all of which complicate standardization (Gao and Gu, 2021; Sadhana et al., 2021). Future research should focus on optimizing carrier materials, improving biosafety evaluation of functional microorganisms, and conducting field validation studies to enhance practical applications. Developing cost-effective and environmentally sustainable solutions will be crucial for integrating microbial immobilization into sustainable agriculture as a viable tool for organophosphorus degradation and soil restoration.

## 5 Conclusion

The incorporation of adsorbent materials ATP and BC with SA had equally positive effects on the adsorption and degradation of phoxim. The addition of ATP or BC adsorbents to the immobilized material SA resulted in the formation of rough honeycomb-like and flake-like structures, as well as special functional groups in the immobilized beads. This was beneficial for the immobilization stability of strain RL4 and improved the adsorption and degradation of phoxim. The SA + ATP-immobilized strain RL4 maintained high degradation activity in agricultural wastewater contaminated with phoxim, making it suitable for *in situ* remediation of this insecticide in polluted environments.

## Data Availability

The original contributions presented in the study are included in the article/Supplementary material, further inquiries can be directed to the corresponding authors.

## Supplementary material

SUPPLEMENTARY FIGURE S1 Scanning electron microscopy of the tested ATP and BC.

SUPPLEMENTARY FIGURE S2 Three types of immobilized beads.

SUPPLEMENTARY TABLE S1 BET adsorption-desorption isotherms and pore size distribution of SA-RL4.

SUPPLEMENTARY TABLE S2 BET adsorption-desorption isotherms and pore size distribution of SA + BC-RL4.

SUPPLEMENTARY TABLE S3 BET adsorption-desorption isotherms and pore size distribution of SA + ATP-RL4.
