# Supplementary figures and images for: Enhanced phoxim biodegradation by immobilizing Novosphingobium sp. RL4 on attapulgite-sodium alginate
Source: Front Microbiol. 2025 Apr 10;16:1541328. doi: 10.3389/fmicb.2025.1541328 (PMC12018411; doi:10.3389/fmicb.2025.1541328)

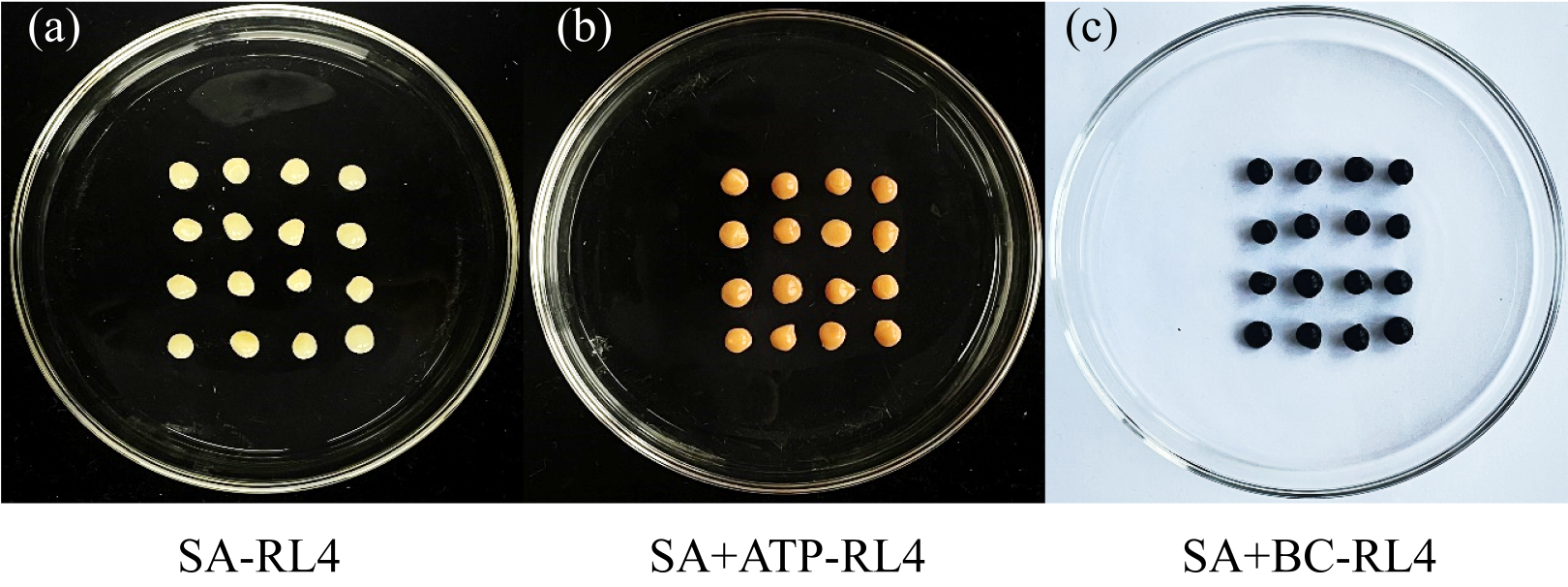

Supplement: SUPPLEMENTARY FIGURE S1 — Scanning electron microscopy of the tested ATP and BC. [file Image_1.TIF]

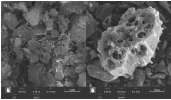

Supplement: SUPPLEMENTARY FIGURE S2 — Three types of immobilized beads. [file Image_2.TIF]

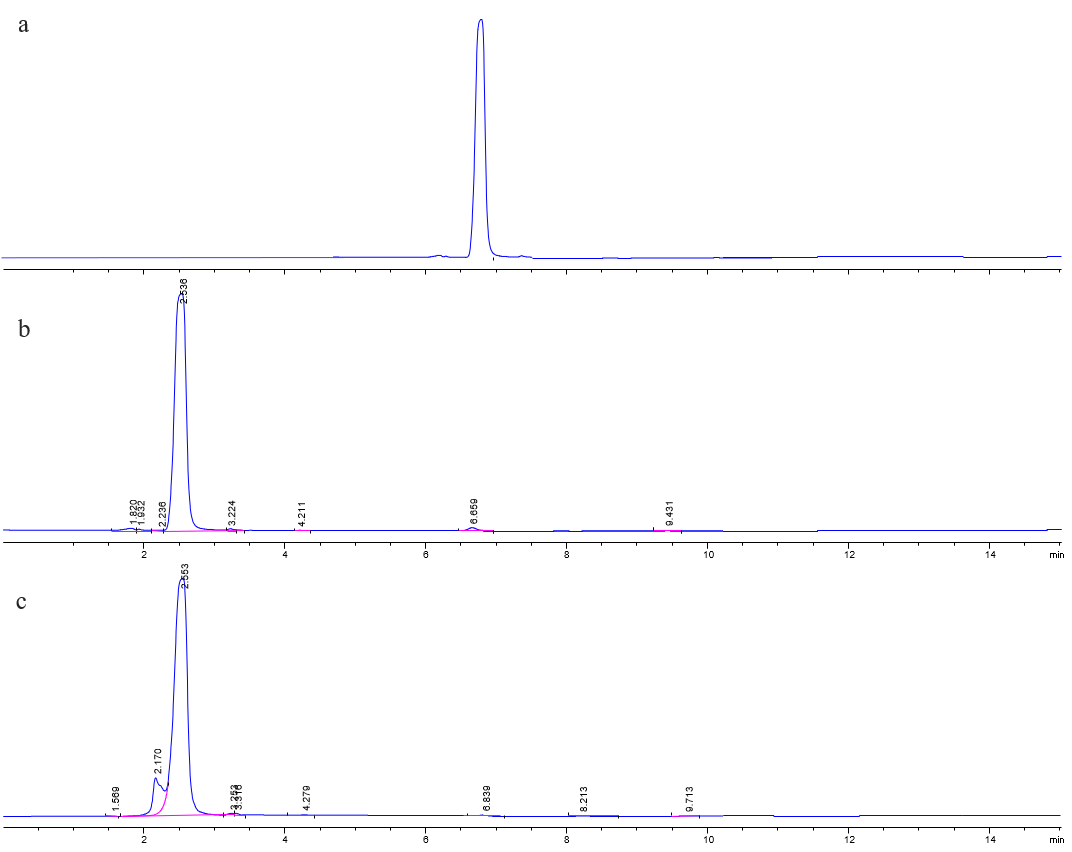

Supplement: Supplementary file 6 [file Data_Sheet_4.ZIP › Source data1/degradation(Figure7-9)/液相.tif]

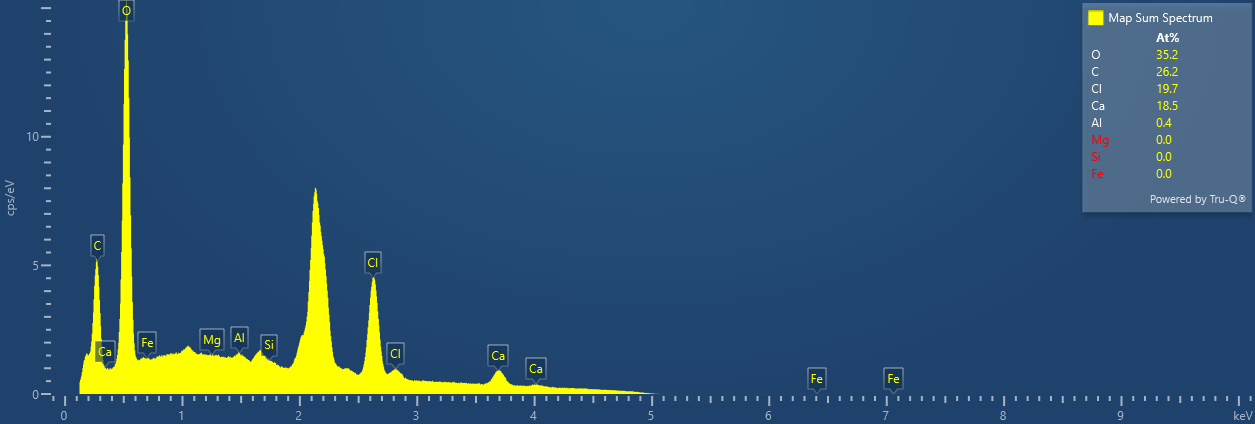

Supplement: Supplementary file 6 [file Data_Sheet_4.ZIP › Source data1/EDS/EDS_Figure 4A.png]

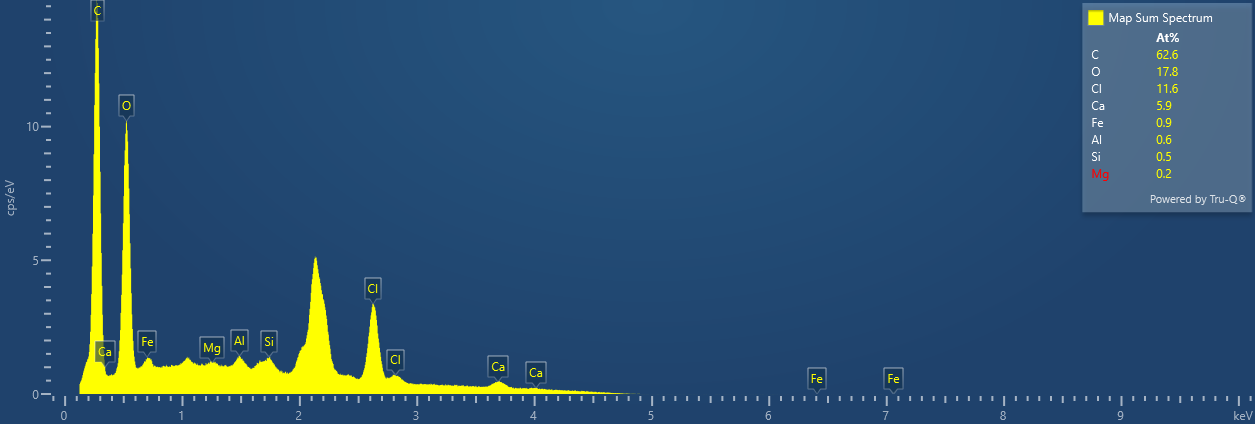

Supplement: Supplementary file 6 [file Data_Sheet_4.ZIP › Source data1/EDS/EDS_Figure 4B.png]

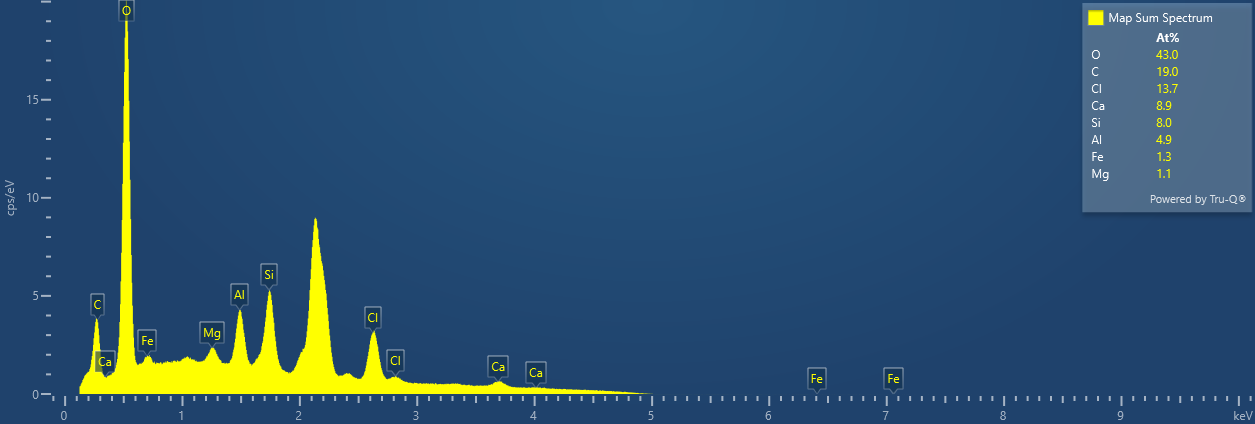

Supplement: Supplementary file 6 [file Data_Sheet_4.ZIP › Source data1/EDS/EDS_Figure 4C.png]

# Bravo Analysis Report

仪器序列号 100648

操作者 Admin

测量日期与时间 18/05/2023 02:55:29 (GMT+0)

## 光谱

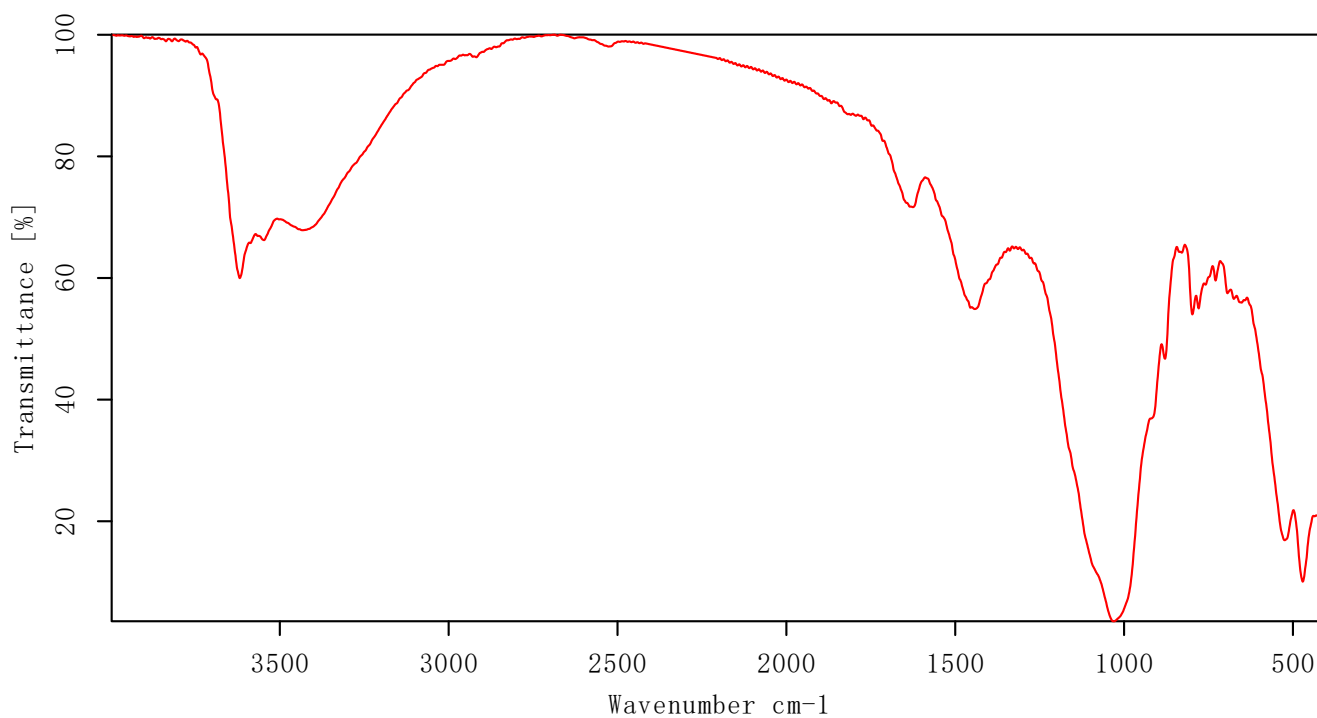

Operator

Review

Supplement: Supplementary file 6 [file Data_Sheet_4.ZIP › Source data1/FTIR/ATP.pdf]

# Bravo Analysis Report

仪器序列号 100648

操作者 Admin

测量日期与时间 18/05/2023 02:43:58 (GMT+0)

## 光谱

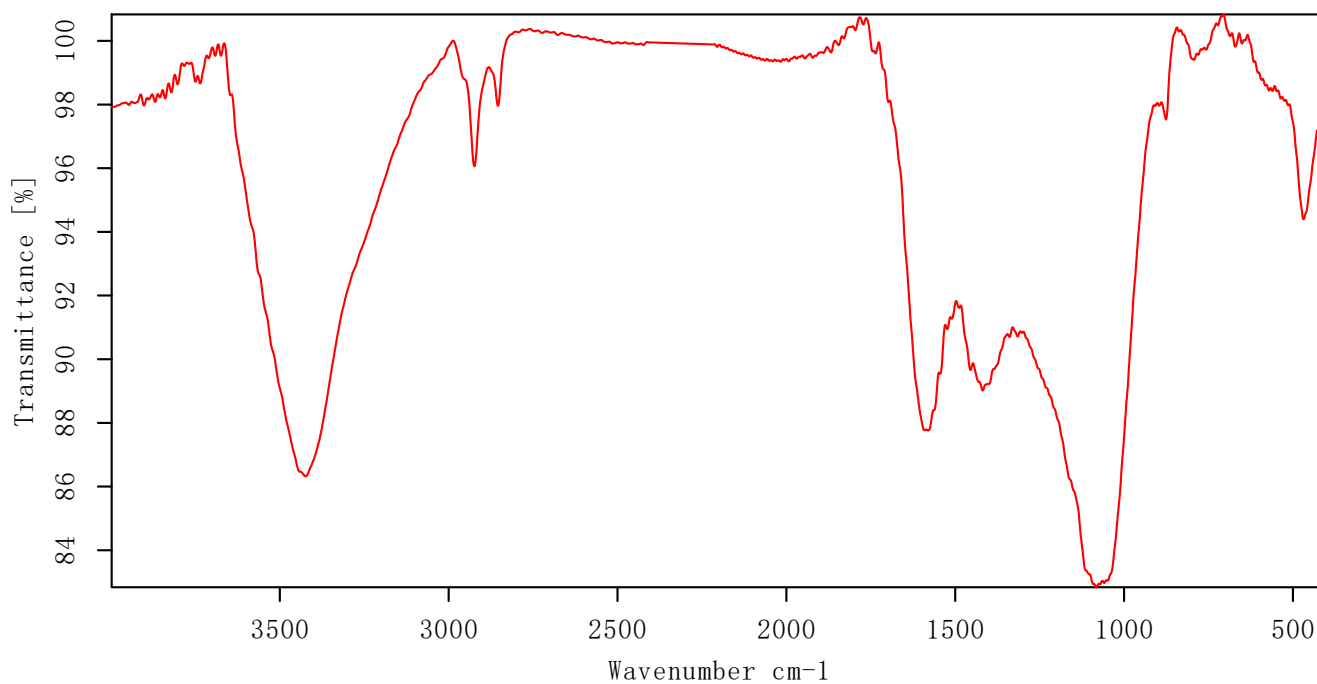

---

Operator

---

Review

Supplement: Supplementary file 6 [file Data_Sheet_4.ZIP › Source data1/FTIR/BC.pdf]

# Bravo Analysis Report

仪器序列号 100648

操作者 Admin

测量日期与时间 18/05/2023 02:01:13 (GMT+0)

## 光谱

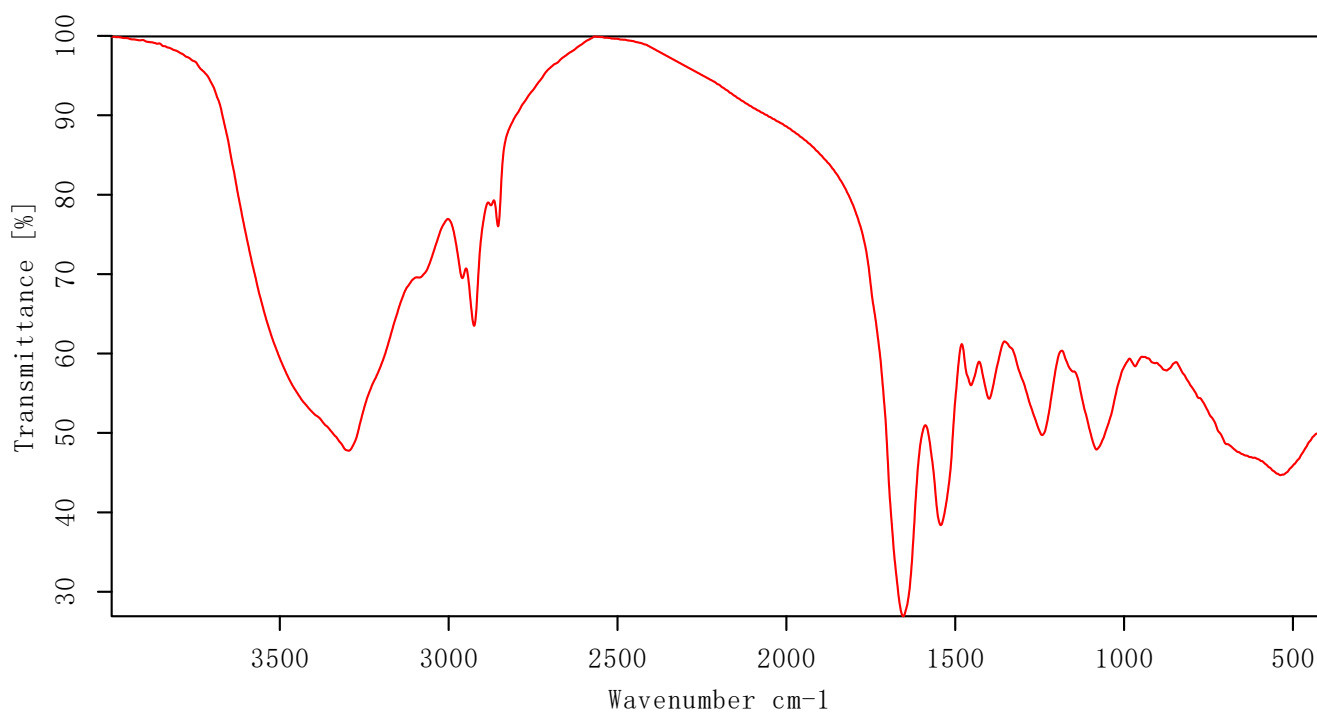

Operator

Review

Supplement: Supplementary file 6 [file Data_Sheet_4.ZIP › Source data1/FTIR/RL4.pdf]

# Bravo Analysis Report

仪器序列号 100648

操作者 Admin

测量日期与时间 18/05/2023 03:35:51 (GMT+0)

## 光谱

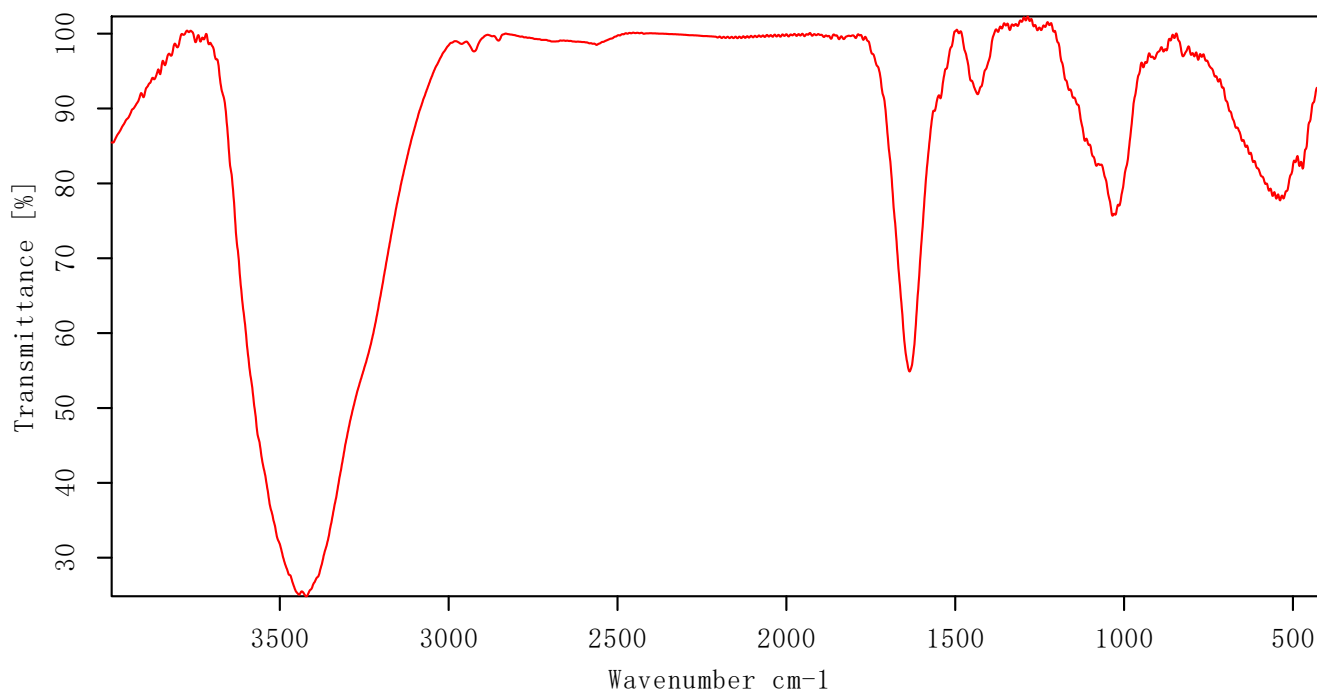

---

Operator

---

Review

Supplement: Supplementary file 6 [file Data_Sheet_4.ZIP › Source data1/FTIR/SA+ATP-RL4.pdf]

# Bravo Analysis Report

仪器序列号 100648

操作者 Admin

测量日期与时间 18/05/2023 03:24:53 (GMT+0)

## 光谱

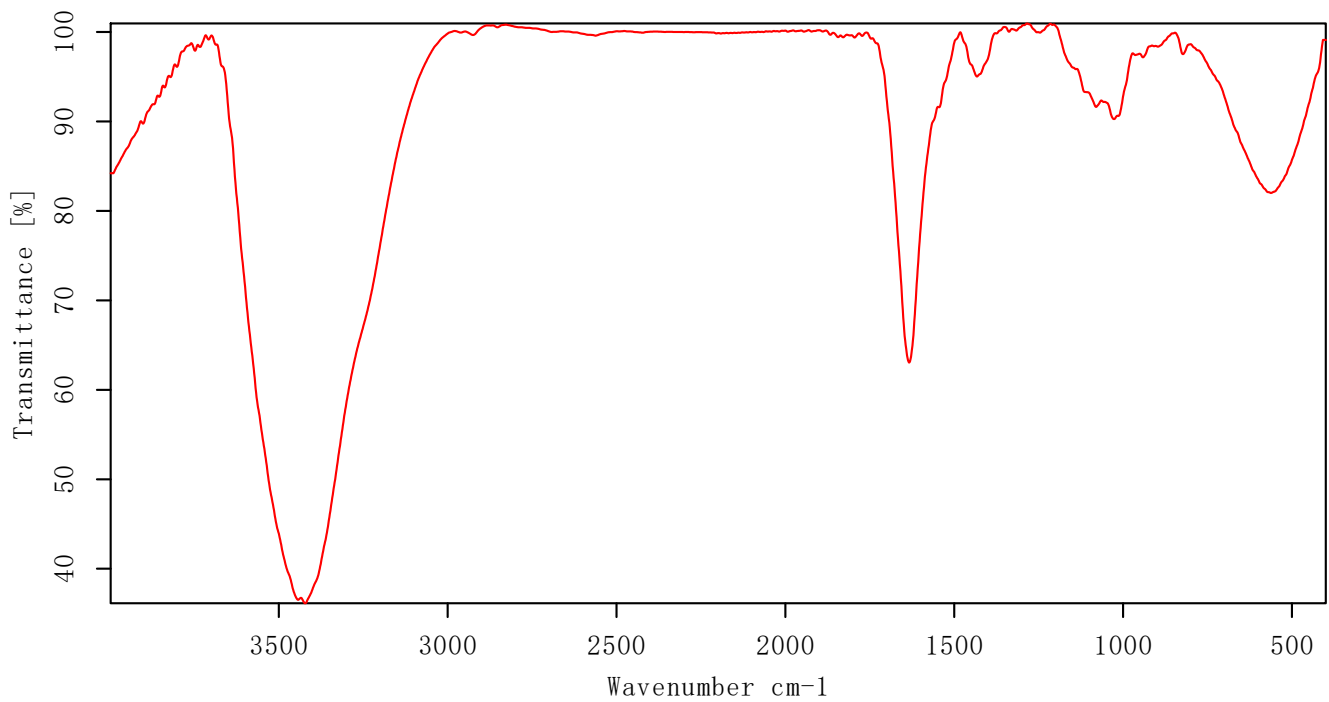

---

Operator

---

Review

Supplement: Supplementary file 6 [file Data_Sheet_4.ZIP › Source data1/FTIR/SA+BC-RL4.pdf]

# Bravo Analysis Report

仪器序列号 100648

操作者 Admin

测量日期与时间 18/05/2023 03:13:20 (GMT+0)

## 光谱

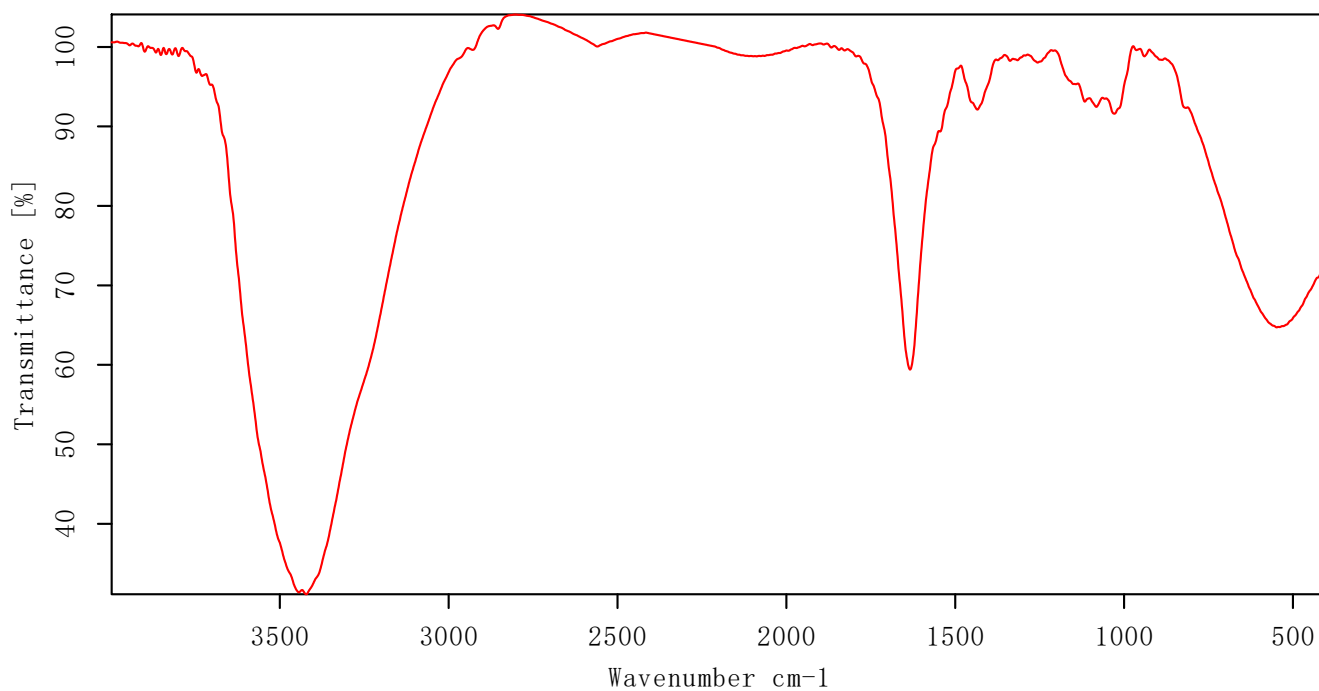

---

Operator

---

Review

Supplement: Supplementary file 6 [file Data_Sheet_4.ZIP › Source data1/FTIR/SA-RL4.pdf]

# Bravo Analysis Report

仪器序列号 100648

操作者 Admin

测量日期与时间 18/05/2023 02:21:53 (GMT+0)

## 光谱

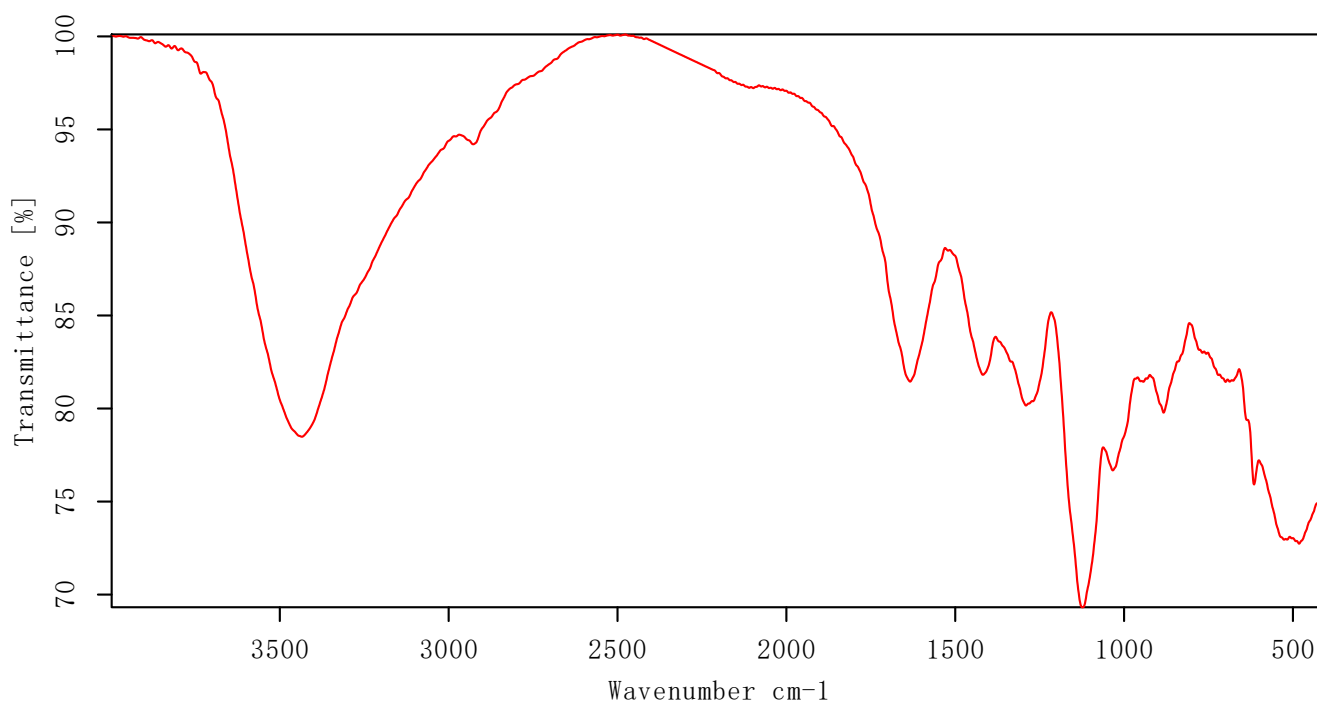

Operator

Review

Supplement: Supplementary file 6 [file Data_Sheet_4.ZIP › Source data1/FTIR/SA.pdf]

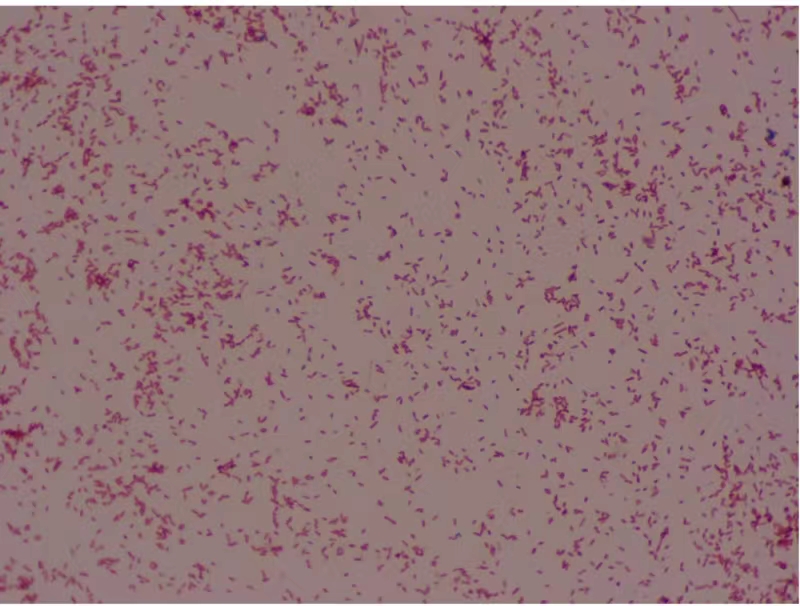

Supplement: Supplementary file 6 [file Data_Sheet_4.ZIP › Source data1/Identification of strain RL4/gram.jpg]

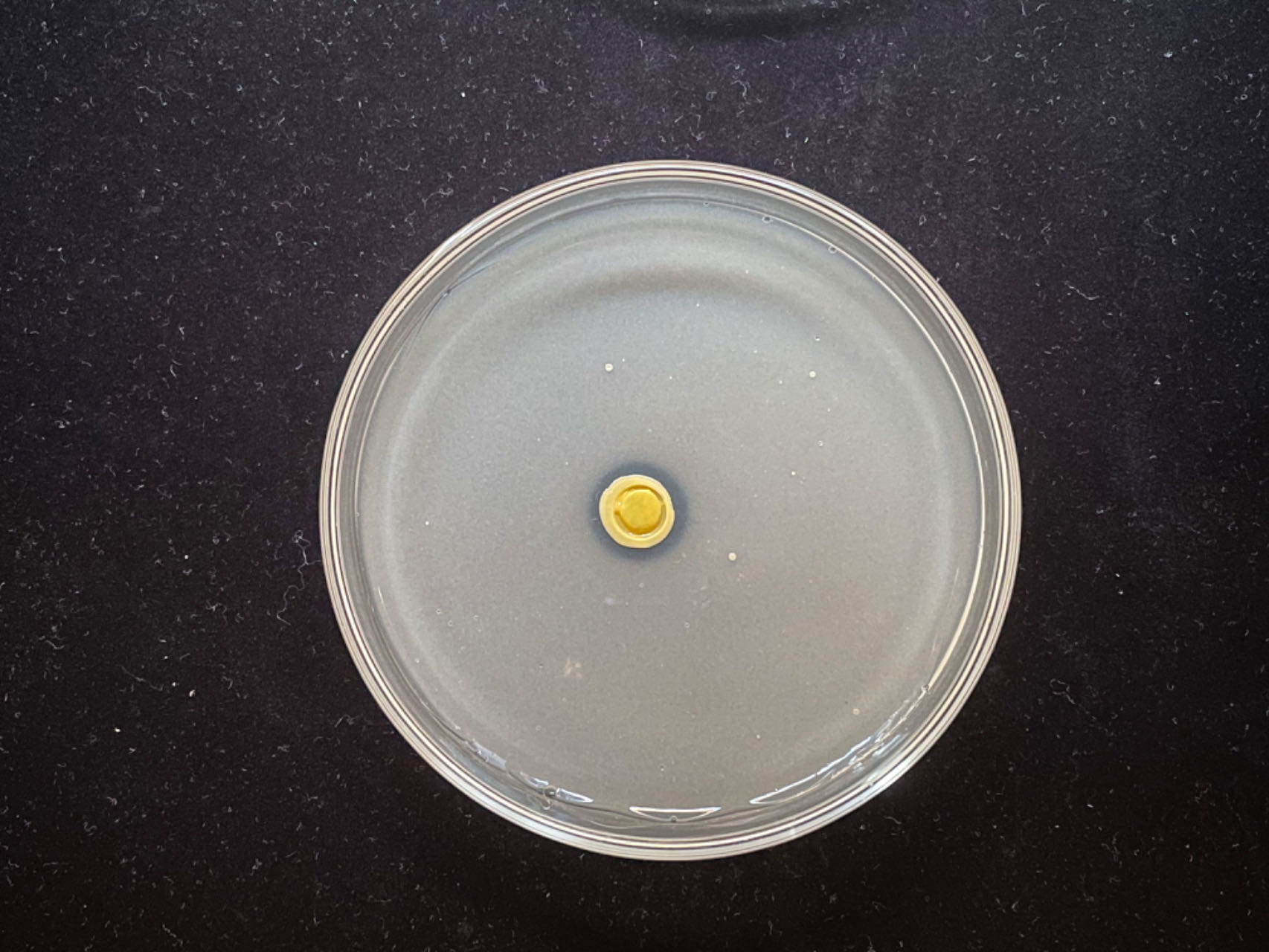

Supplement: Supplementary file 6 [file Data_Sheet_4.ZIP › Source data1/Identification of strain RL4/RL4 strain-phoxim hydrolysis circle.jpg]

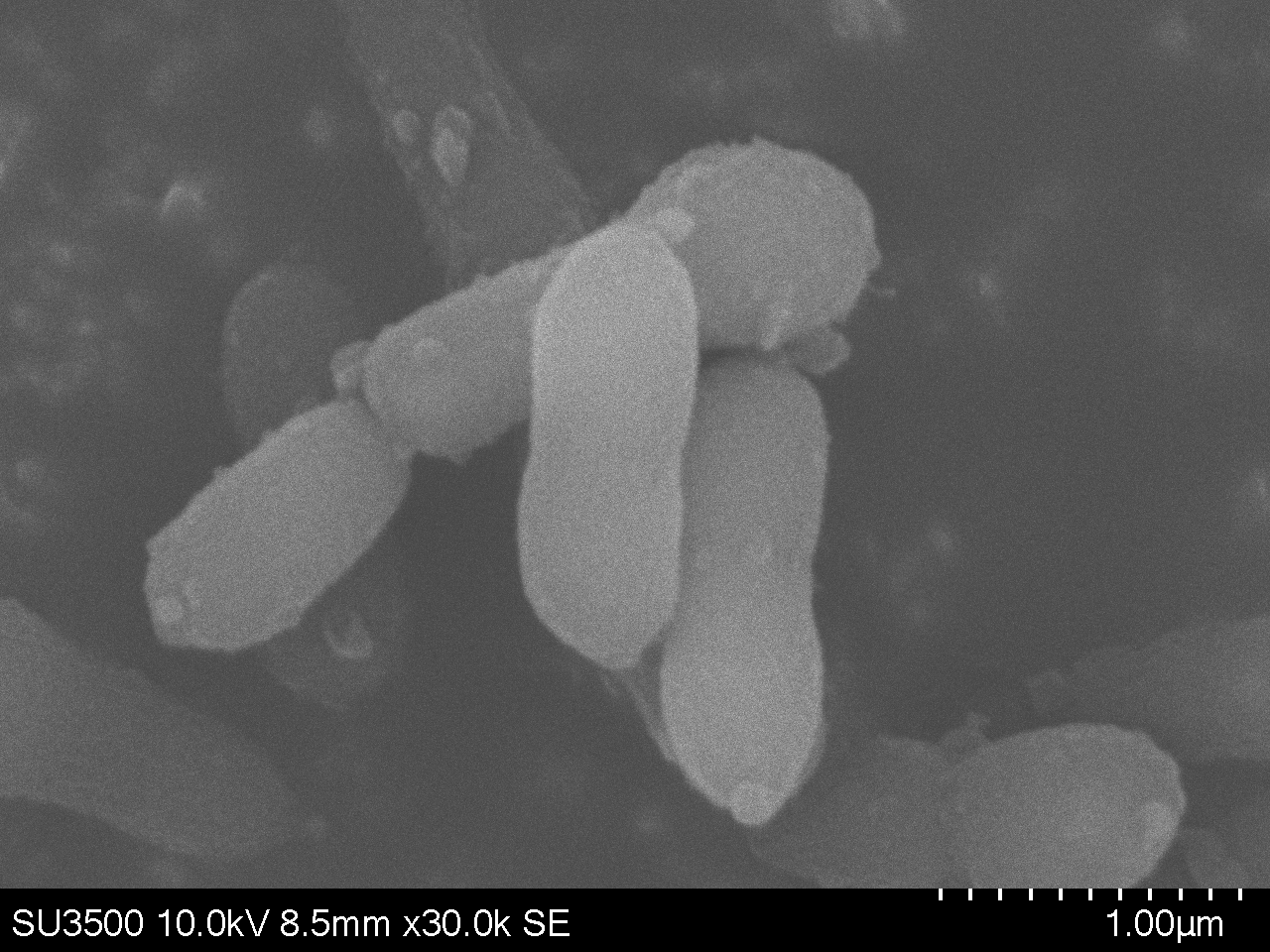

Supplement: Supplementary file 6 [file Data_Sheet_4.ZIP › Source data1/Identification of strain RL4/SEM.tif]

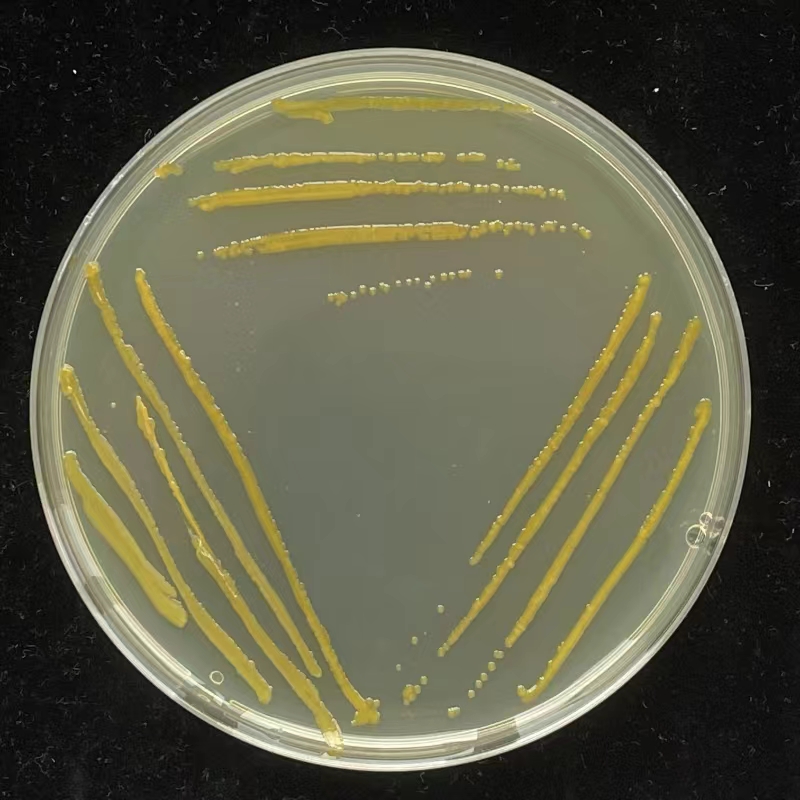

Supplement: Supplementary file 6 [file Data_Sheet_4.ZIP › Source data1/Identification of strain RL4/strain morphology of LB.jpg]
